# Lack of a significant impact of Gag-Protease-mediated HIV-1 replication capacity on clinical parameters in treatment-naive Japanese individuals

**DOI:** 10.1186/s12977-015-0223-z

**Published:** 2015-11-19

**Authors:** Keiko Sakai, Takayuki Chikata, Zabrina L. Brumme, Chanson J. Brumme, Hiroyuki Gatanag, Shinichi Oka, Masafumi Takiguchi

**Affiliations:** Center for AIDS Research, Kumamoto University, Kumamoto, 860-0811 Japan; International Research Center for Medical Sciences, Kumamoto University, Kumamoto, 860-0811 Japan; British Columbia Centre for Excellence in HIV/AIDS, Vancouver, BC Canada; Faculty of Health Sciences, Simon Fraser University, Burnaby, BC V5A 1S6 Canada; National Center for Global Health and Medicine, Tokyo, 162-8655 Japan; Nuffield Department of Medicine, University of Oxford, Oxford, UK

**Keywords:** HIV-1, Replication capacity, Gag, Fitness

## Abstract

**Background:**

HLA class I-associated escape mutations in HIV-1 Gag can reduce viral replication, suggesting that associated fitness costs could impact HIV-1 disease progression. Previous studies in North American and African cohorts have reported reduced Gag-Protease mediated viral replication capacity (Gag-Pro RC) in individuals expressing protective HLA class I alleles including HLA-B*57:01, B*27:05, and B*81:01. These studies also reported significant positive associations between Gag-Pro RCs and plasma viral load (pVL). However, these HLA alleles are virtually absent in Japan, and the importance of Gag as an immune target is not clearly defined in this population.

**Results:**

We generated chimeric NL4-3 viruses carrying patient-derived Gag-Protease from 306 treatment-naive Japanese individuals chronically infected with HIV-1 subtype B. We analyzed associations between Gag-Pro RC and clinical markers of HIV-1 infection and host HLA expression. We observed no significant correlation between Gag-Pro RC and pVL in Japan in the overall cohort. However, upon exclusion of individuals expressing Japanese protective alleles HLA-B*52:01 and B*67:01, Gag-Pro RC correlated positively with pVL and negatively with CD4 T-cell count. Our results thus contrast with studies from other global cohorts reporting significantly lower Gag-Pro RC among persons expressing protective HLA alleles, and positive relationships between Gag-Pro RC and pVL in the overall study populations. We also identified five amino acids in Gag-Protease significantly associated with Gag-Pro RC, whose effects on RC were confirmed by site-directed mutagenesis. However, of the four mutations that decreased Gag-Pro RC, none were associated with reductions in pVL in Japan though two were associated with lower pVL in North America.

**Conclusions:**

These data indicate that Gag fitness does not affect clinical outcomes in subjects with protective HLA class I alleles as well as the whole Japanese population. Moreover, the impact of Gag fitness costs on HIV-1 clinical parameters in chronic infection is likely low in Japan compared to other global populations.

**Electronic supplementary material:**

The online version of this article (doi:10.1186/s12977-015-0223-z) contains supplementary material, which is available to authorized users.

## Background

Within an infected individual, immune pressure by cytotoxic T lymphocytes (CTLs) induces the selection of HLA-associated escape variants in the HIV-1 genome. The persistence of HLA-associated escape variants upon transmission in turn shapes viral diversity at the population level [1]. CTL escape occurs throughout infection. Moreover, certain escape mutations compromise viral fitness. In some of these cases, their effects on immune evasion can be offset in part by their fitness costs though the effects of such mutations on plasma viral load (pVL) are complex and remain incompletely understood [2–9]. HIV-1 Gag is rich in immunodominant CTL epitopes, and a vital role of anti-Gag CTL responses on HIV-1 immune control has been demonstrated [10, 11; also reviewed in 12, 13]. In particular, associations between fitness-costly Gag escape mutations and decreases in pVL have been described [2, 5–8], notably for the immunodominant HLA-B*57:01-restricted TW10 and B*27:05-restricted KK10 epitopes, both of which are well studied because of their protective roles in HIV-1 disease outcome. Specifically, CTL-driven escape mutations in TW10 impose a mild fitness cost that, along with robust HLA-B*57:01-driven CTL responses, likely contribute to the significantly lower pVL observed in these subjects [5, 7, 14–17]. Notably however, fitness costs of escape in HLA-B*57:01 epitopes are generally rescued by the subsequent appearance of compensatory mutations [18, 19]. In contrast, a single amino acid change at Gag 264 (R264K) in the B*27:05-restricted KK10 epitope substantially compromises in vitro viral replication [20–22], but this mutation generally occurs late in infection due to the requirement for the upstream compensatory mutation S173A [7, 21]. As such, the appearance of R264K in vivo generally heralds HIV-1 progression [23, 24]. These studies support a relationship between Gag escape mutations and pVL and underscore a critical role of Gag in HIV-1 disease outcome.

In vitro studies also highlight the importance of Gag as an immune target. Certain HLA-associated mutations in Gag have been shown to impair Gag-Protease-mediated viral replication capacity in vitro (Gag-Pro RC). Gag-Pro RC is in turn positively associated with pVL in numerous global cohorts [18, 25–30]. In particular, reduced Gag-Pro RC has been observed in predominantly North American cohorts of elite controllers as well as individuals in acute/early infection who express protective alleles B*13, B*27, B*57, and/or B*58:01 [18, 26], though the latter associations are less apparent during the chronic phase [18]. In addition, the impact of HLA-associated changes in Gag-Pro RC on pVL have also been reported in African cohorts chronically infected with HIV-1 subtype C [27–29]. A more recent study compared Gag-Pro RCs in treatment-naive Mexican and Barbadian HIV-1 subtype B cohorts which differ in their frequency of protective HLA alleles (8 % versus 34 % of B*27/57/58:01/81:01 in Mexico versus Barbados). The study revealed a higher burden of HLA-mediated Gag escape mutations and significantly lower Gag-Pro RC in chronically infected Barbadian compared to Mexican individuals [30]. Moreover, Gag-Pro RC correlated positively with pVL, supporting the importance of Gag in HIV-1 disease outcome [30]. The significantly lower Gag-Pro RC and pVL in Barbados versus Mexico was attributed to the higher prevalence of protective HLA alleles in the former cohort, implying that protective alleles lower Gag-Pro RC, viral set points, and therefore, disease progression rates at the population level. These results are consistent with a previous study of nine distinct cohorts worldwide that demonstrates HIV-1 adaptation to HLA class I alleles at a population level, presumably as a result of transmission and persistence of certain escape mutations in the population [1]. Thus, HLA allele frequency shapes viral diversity, which could also affect CTL responses and HIV-1 disease outcome in the population.

HLA class I distributions differ markedly in Asian compared with North American and African cohorts [31]. The “classical” protective alleles B*27:05 and B*57:01 are essentially absent in Japan: a published cohort of 504 treatment-naive HIV^+^ Japanese featured only one B*27:05^+^ and no B*57:01 patients [31–37]. Instead, B*52:01 and B*67:01 are associated with HIV-1 control in Japan [32]. Therefore, the role of Gag fitness in HIV-1 pathogenesis, or more broadly the mechanism of protection, is likely to be different in Asian populations. As such, the impact of HLA-mediated immune pressure on viral fitness and its influence on HIV-1 pathogenesis need to be specifically defined for Asian populations. To address these issues, we investigated the effect of HLA-associated changes in Gag-Pro RC in 306 treatment-naive HIV^+^ Japanese patients chronically infected with HIV-1 subtype B in the presence or absence of protective HLA alleles. We also identified amino acid changes in Gag-Protease that altered Gag-Pro RCs and examined their associations with pVL. In contrast to studies of non-Asian cohorts that supported a role of Gag fitness in HIV-1 disease outcome, our results suggested that Japanese protective alleles did not lower Gag-Pro RC and that Gag fitness did not correlate with clinical markers in the whole Japanese population.

## Results

### Relationship between Gag-Protease-dependent replication capacity and clinical markers of disease progression

**Fig. 1:**
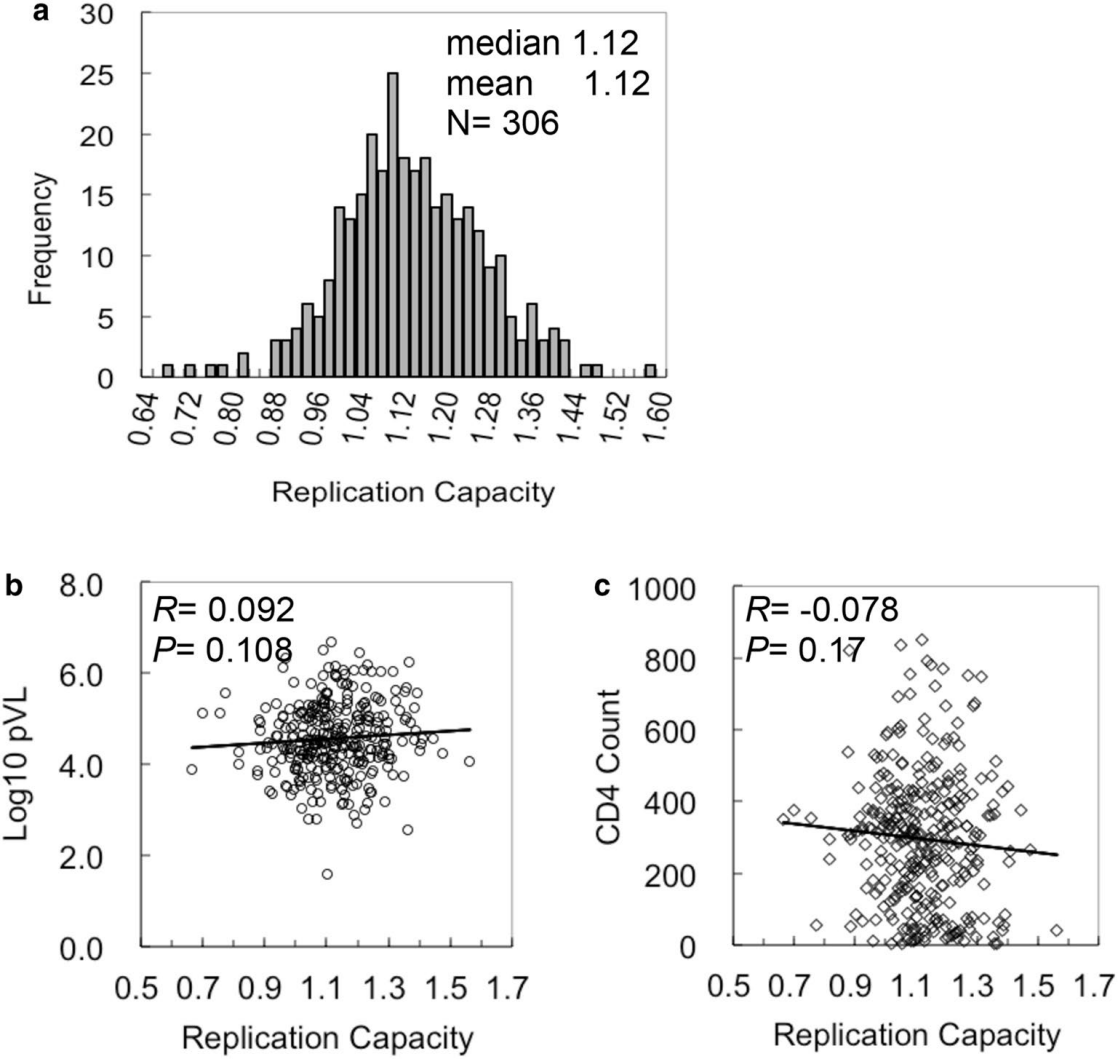
Gag-mediated replication capacities in a treatment naive Japanese cohort chronically infected with HIV-1 subtype B. **a** Distribution of Gag-Protease replication capacities. Recombinant HIV-1_NL4-3_ was constructed, using plasma-derived *gag-protease* from treatment-naïve HIV^+^ Japanese patients, and replication capacity for each virus was determined by in vitro infection. The values were normalized against mean growth rate of wild-type NL4-3. The samples were measured in triplicate. **b**, **c** Replication capacities and patients’ clinical parameters. Replication capacities of chimeric viruses were plotted against plasma viral load (**b**) or CD4 count (**c**) at the time of sample collection. Correlations were determined by Spearman’s rank correlation test

We generated recombinant HIV-1_NL4-3_ containing Gag-Protease from 306 treatment-naïve Japanese subjects chronically infected with HIV-1 subtype B. Plasma viral sequences were determined for all patients in a previous study [31], and the patient origin of all recombinant viruses was confirmed by maximum likelihood phylogenetic analysis (Additional file 1). Recombinant viruses were used to infect a reporter CD4^+^ T-cell line, CEM-GXR, which naturally expresses CXCR4 and has been further engineered to express CCR5 and Tat-inducible GFP [38]. Gag-Protease-dependent replication capacities (Gag-Pro RCs) were determined based on the exponential increase in the  % GFP^+^ cells during days 2–8 following infection and normalized against that of the parental HIV-1_NL4-3_ as described for North American and African studies [18, 25–30]. Similar to previous reports, Gag-Pro RCs were normally distributed (Fig. 1a), with a mean of 1.12, a standard deviation (SD) of 0.13, and a range of 0.67–1.56. The mean Gag-Pro RC in the Japanese cohort was slightly higher than that observed in the North American cohort [18] but comparable to those reported more recently for an ART-naïve Mexican cohort [30].

To examine the impact of Gag-Pro RC on HIV-1 pathogenesis, we investigated the relationship between Gag-Protease RC and clinical markers of HIV-1 infection (pVL and CD4 T-cell count). Overall, Japanese Gag-Pro RCs did not correlate with pVL (*R* = 0.092, *P* = 0.11; Fig. 1b) nor CD4 T-cell count (*R* = −0.078, *P* = 0.17; Fig. 1c). Not only did these results not achieve statistical significance, but the correlation coefficients for pVL and Gag-Pro RC in Japan were weaker than those reported in African (*R* = 0.24) [28, 29] and Mexican/Barbadian (*R* = 0.25) [30] chronic cohorts. The Japanese correlation coefficient *R* = 0.092 was also weaker to that reported in a North American chronic cohort (*R* = 0.27 in the presence of protective HLA alleles; *R* = 0.12 for the total population) [18]. Together, this result suggested a minimal role for Gag fitness as a major determinant of virologic control in Japan compared to other global cohorts.

### Impact of HLA class I alleles on Gag-Pro RCs and relationship with clinical parameters

To investigate the role of Gag as an HLA-mediated immune target, we stratified HLA class I alleles based on their median RCs (Additional file 2A-C). For all alleles with frequencies >3 % (n > 8) in the cohort, we did not find any significant associations between HLA alleles and Gag-Pro RC. In addition, for each HLA allele, we compared the relative rankings of median RCs amongst the HLA alleles with those previously determined for pVL [32]. If Gag-Pro RC affects pVL, HLA alleles with lower Gag-Pro RCs would show a tendency toward lower pVL and vice versa. However, the relative RC ranking (in Additional file 2A-C) and pVL for each HLA allele did not match in most cases. We previously reported that A*33:03, B*44:03, B*40:06, C*14:03, and C*12:02 were associated with lower pVL in Japan [32], but none of these alleles were associated with lower Gag-Pro RC (Additional file 2A-C). Notably, the median Gag-Pro RCs in individuals expressing Japanese protective alleles HLA-B*52:01 and B*67:01 [32] were higher than the population median (Additional file 2B), indicating that protective-allele expression is not associated with lower Gag fitness in Japan. Indeed, no significant correlation was observed between median Gag-Pro RCs and median pVL for each HLA allele (Additional file 2D).

**Fig. 2:**
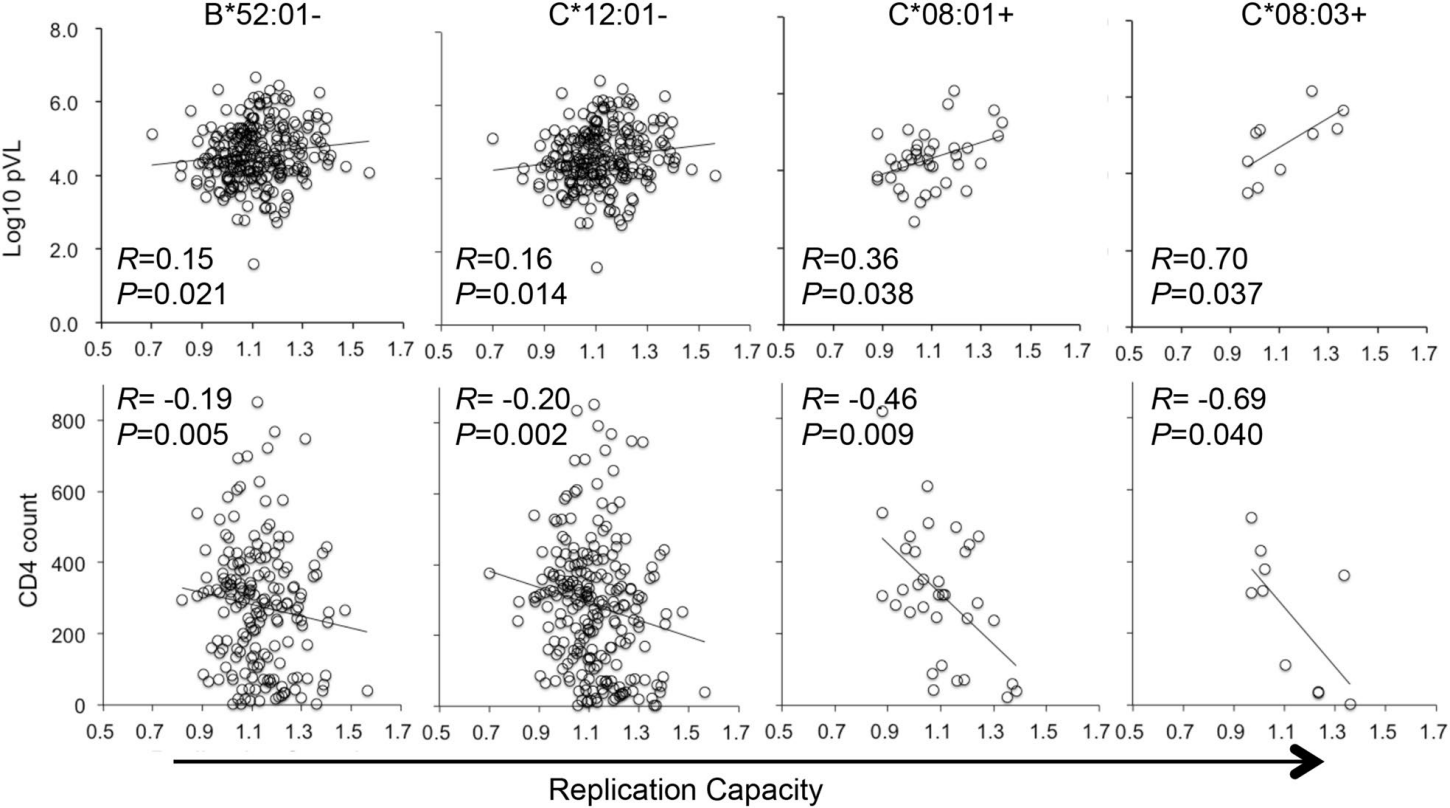
Replication capacities for HLA alleles and their associations with clinical parameters. The presence (HLA-C*08:01 and C*08:03) or absence (HLA-B*52:01 and C*12:01) of four HLA alleles showed significant associations with pVL (*top panels*) as well as CD4 count (*bottom panels*). The associations were examined by Sperman’s rank correlation test

To further explore the relationship between HLA, Gag-Pro RC, and HIV-1 clinical parameters, we grouped our cohort by HLA-allele expression and analyzed associations between Gag-Pro RCs and HIV-1 clinical parameters in each HLA group (Table 1). In two HLA-A alleles (A*02:01 and A*31:01), one B allele (B*07:02), and two C alleles (C*08:01 and C*08:03), Gag-Pro RC exhibited a positive correlation with pVL (p < 0.05), whereas Gag-Pro RC exhibited negative correlations with CD4 count at this threshold in seven alleles (HLA-A*02:06, HLA-B*46:01, B*48:01, B*59:01, C*01:02, C*08:01, and C*08:03; Table 1). However, only in HLA-C*08:01^+^ (n = 34) and C*08:03^+^ (n = 10) subjects, Gag-Pro RC correlated with both clinical parameters: a positive correlation with pVL (*R* = 0.36 and *R* = 0.70, respectively) and an inverse correlation with CD4 count (*R* = −0.46 and *R* = −0.69, respectively; Fig. 2).

**Table 1:** Associations of Gag-Pro RCs with clinical parameters in each HLA class I allele

Associations with pVL						Associations with CD4 count					
HLA allele	HLA + or −^a^	n	R	*P* value	*q* value	HLA allele	HLA + or −^a^	n	R	*P* value	*q* value
A*02:01	+	63	0.26	0.0428	0.243	A*02:06	+	61	−0.29	0.0231	0.279
A*31:01	+	48	0.31	0.0357	0.238	B*46:01	+	31	−0.41	0.0264	0.279
B*07:02	+	24	0.46	0.0288	0.238	B*48:01	+	14	−0.65	0.0194	0.279
C*08:01	+	34	0.36	0.0377	0.238	B*59:01	+	14	−0.59	0.0344	0.318
C*08:03	+	10	0.70	0.0365	0.238	C*01:02	+	92	−0.28	0.0082	0.163
A*11:01	−	252	0.14	0.0277	0.238	C*08:01	+	34	−0.46	0.0088	0.163
A*33:03	−	265	0.14	0.0288	0.238	C*08:03	+	10	−0.69	0.0399	0.328
B*39:01	−	285	0.12	0.0385	0.238	B*52:01	−	234	−0.19	0.0045	0.163
B*44:03	−	266	0.14	0.0244	0.238	C*12:02	−	231	−0.20	0.0024	0.163
B*52:01	−	234	0.15	0.0209	0.238						
C*07:02	−	233	0.14	0.0325	0.238						
C*12:02	−	231	0.16	0.0135	0.238						
C*14:03	−	267	0.14	0.0251	0.238						

Italic HLA alleles have associations with both pVL and CD4 count

*n* number of subjects, *R* Spearman’s rank correlation

^a^HLA+ and HLA− indicate subjects with or without the particular HLA allele, respectively

We also examined groups of patients based on non-expression of particular HLA alleles. We observed significant correlations between clinical parameters and Gag-Pro RCs in the absence of eight HLA alleles (Table 1), including HLA-B*52:01. In individuals lacking the Japanese protective B*52:01 allele, Gag-Pro RCs correlated positively with pVL (*R* = 0.15, *P* = 0.021, q = 0.24, n = 234) and inversely with CD4 T-cell count (*R* = −0.19, *P* = 0.005, q = 0.16; Fig. 2). Similarly, Gag-Pro RC also showed significant associations with both pVL and CD4 count in individuals lacking HLA-C*12:02 (Fig. 2). HLA-B*52:01 and C*12:02 form a haplotype in Japan [31–36]. Indeed, all B*52:01^+^ subjects had C*12:02, and of 85 C*12:02^+^ subjects in our cohort, only three did not carry B*52:01. Positive correlations between Gag-Pro RC and pVL were additionally observed in the absence of A*11:01, A*33:03, B*39:01, B*44:03, C*07:02, and C*14:03 (Table 1), but none of these alleles associated with CD4 count. Taken together, our results stand in contrast to observations of lower Gag-Pro RC among individuals expressing classical protective HLA alleles in other global cohorts [18, 25–30]. HIV-1 control by protective HLA alleles is not associated with low Gag fitness in Japan.

### Impact of Gag on clinical parameters in the absence of protective alleles

**Fig. 3:**
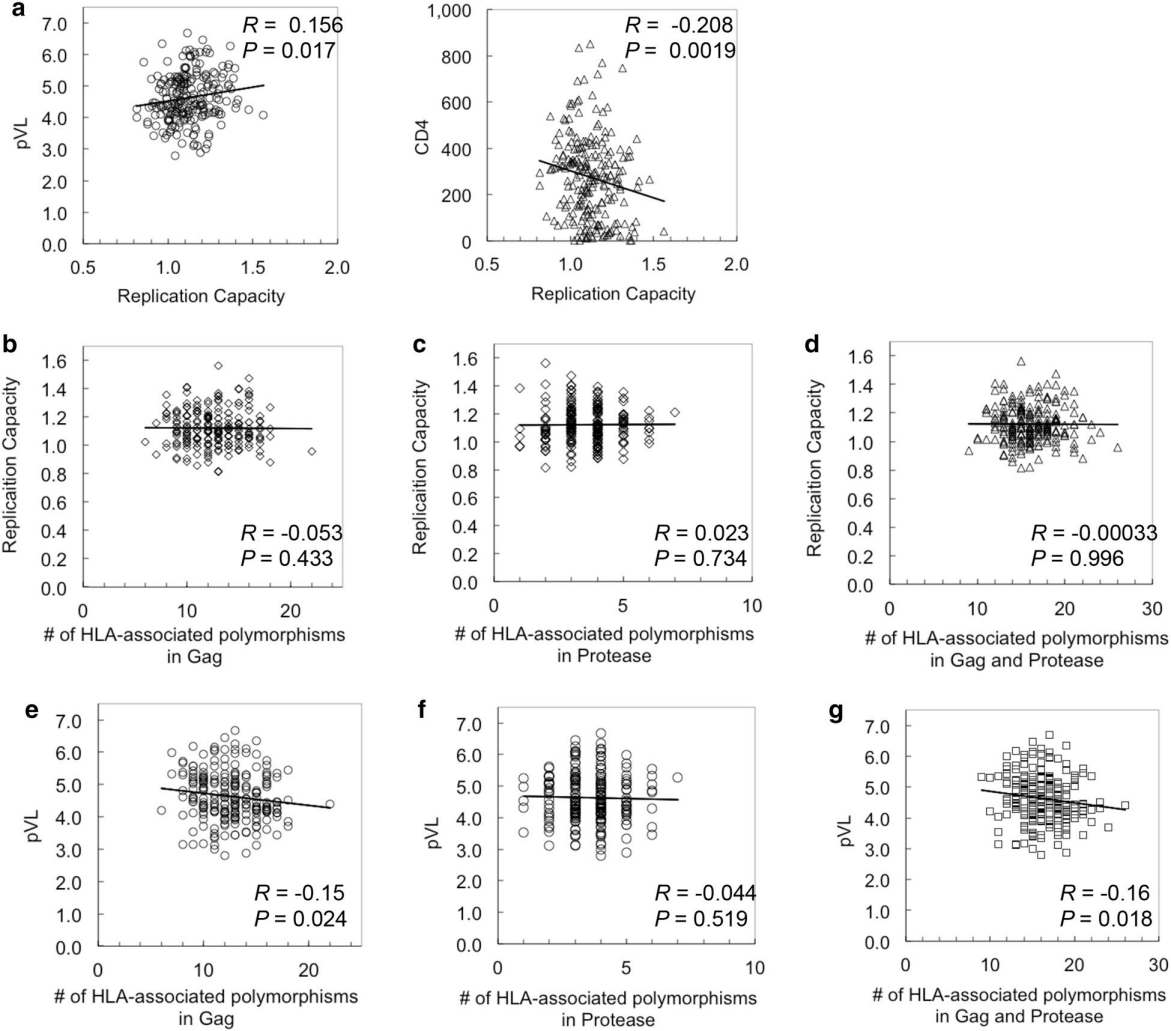
The relationship between HLA-associated-polymorphisms and Gag-Protease-mediated replication capacities in the HLA-B*52:01^−^B67:01^−^ population. **a** Associations of replication capacities with plasma viral load and CD4 count in the B*52:01^−^B*67:01^−^ population were tested by Spearman’s rank correlation test. **b**–**g** An overall number of HLA-induced mutations in Gag (**b**), Protease (**c**), and both regions (**d**) were plotted against Gag-Protease-mediated replication capacities to assess their impact on viral fitness. Their effects on patients’ plasma viral load were also tested statistically (**e**–**g**)

In our cohort, Gag-Pro RCs did not correlate with pVL in subjects carrying either of the Japanese protective alleles, B*52:01 or B*67:01. However, exclusion of B*52:01 (n = 234) resulted in associations of Gag-Pro RCs with clinical markers of infection (*R* = 0.15, *P* = 0.021, *q* = 0.24 for pVL; Table 1). Therefore, we next examined correlations between Gag-Pro RCs and clinical parameters in the absence of these protective alleles (i.e. among B*52:01 and B*67:01 double-negative subjects; n = 224). In persons lacking both of these protective alleles, Gag-Pro RCs exhibited a positive association with pVL (*R* = 0.16, *P* = 0.017) and a negative association with CD4 count (*R* = −0.21, *P* = 0.0019) (Fig. 3a). These associations are weaker than those observed in Mexican/Barbadian, African, and North American individuals expressing protective HLA alleles (R > 0.2 for these cohorts) but comparable in magnitude to the relationship between Gag-Pro RC and pVL in the North American chronic cohort (*R* = 0.12, *P* = 0.0007) [18, 29, 30]. Indeed, after excluding B*52/B*67/C*12 alleles, the associations of Gag-Pro RC and clinical parameters became slightly stronger in each HLA allele^-^ group analyzed in Table 1 (Additional file 3). These observations further support our result that Gag fitness does not associate with clinical parameters in subjects with protective HLA alleles in Japan.

When we further explored the relationship between Gag-Pro RC and HIV clinical parameters in the B*52:01^−^B*67:01^−^ subpopulation by multivariable analyses, a linear regression model adjusting for pVL and CD4 count indicated that Gag-Pro RC in this subset was driven largely by CD4 count (*P* = 0.043; β Estimate = −0.012 per 100 cell/mm^3^ increment) and not pVL (*P* = 0.47; β Estimate = 0.0091 per log_10_ increment). In other words, the model indicated that, after controlling for pVL, Gag-Pro RC decreased by 0.012 units for each increment of 100 CD4 cells/mm^3^. Although the model identified CD4 count as an independent predictor of Gag-Pro RC, CD4 count explains only 3.7 % of population variation in Gag-Pro RC (Table 2). In another linear regression model additionally incorporating three HLA alleles—C*08:01, C*08:03, and C*12:02—that exhibited significant associations of Gag-Pro RC with both pVL and CD4 count in the B*52:01^−^B*67:01^−^ subpopulation, the addition of HLA information to the model did not improve the model fit (*P* = 0.12). Taken together, results of the multivariable analyses suggest that apparent HLA associations with Gag-Pro RC, HIV clinical parameters, and HLA alleles are likely to be driven primarily by CD4 count rather than pVL in subjects lacking the protective B*52 and B*67 alleles. Moreover, these observations raise the possibility that Gag-Pro RC may not have a major impact on pVL even in the B*52^−^B*67^−^ subpopulation.

**Table 2:** Linear regression models investigating the relationship between Gag-Pro RC and clinical factors (analysis limited to individuals lacking B*52/B*67)

Variable	Multivariable linear regression model parameters (Gag-Pro RC)^a^	
	Estimate	*P* value
Log_10_pVL	9.13 × 10^−3^	0.47
CD4	−1.16 × 10^−4^	*0.043*

β Estimates for log_10_viral load are expressed per log_10_ increment. Estimates for CD4 count are expressed per 1 cell/mm^3^ increment

^a^Model: multiple r^2^ = 0.037, *P* = 0.015

We next analyzed the impact of HLA associated polymorphisms on pVL in 218 B*52:01^−^ B*67:01^−^ subjects based on the HLA associated polymorphisms determined for the Japanese population [31]. Here, HLA-associated polymorphisms were defined based on a published list derived from plasma virus sequences from the present cohort (see methods and [31]; these included 94 HLA-associated polymorphisms in Gag and 16 in Protease). For each individual in this analysis, we counted the number of HLA-associated polymorphisms present in their viral sequence (regardless of the host HLAs expressed). In doing so we observed no correlation between the number of polymorphisms in Gag, Protease, or Gag-Protease and Gag-Pro RCs (Fig. 3b–d). On the other hand, statistically significant negative correlations were observed between the number of HLA-associated polymorphisms in Gag (*R* = −0.15, *P* = 0.024) as well as Gag-Protease (*R* = −0.16, *P* = 0.018) and pVL (Fig. 3e–g). This suggests that HLA-associated polymorphism burden in Gag impacts pVL without significantly affecting Gag-Pro RCs in the B*52^−^B*67^−^ subpopulation, indicating the possible absence of correlation between Gag-Pro RC and pVL in this subpopulation. A simple explanation for the discrepancy would be that a combinatorial effect by mutations in other parts of the virus may have resulted in the significant change in pVL even though the number of polymorphisms in Gag only was not enough to affect Gag replication per se.

### Impact of amino acid changes on Gag RCs in the absence of the protective alleles

**Fig. 4:**
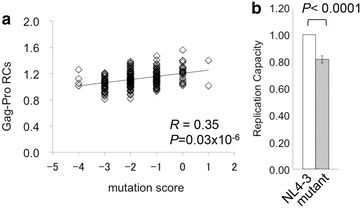
The impact of amino acid polymorphisms in Gag on viral fitness. **a** The x- and y-axes indicate mutation scores and replication capacities, respectively. Mutation scores were calculated by giving −1 for each polymorphism associated with decreased replication capacities in the HLA-B*52:01^−^B*67:01^−^ subpopulation, i.e. at Gag 79, 228, 286, and 357, and +1 for a polymorphism at Gag 218, which was associated with an increased replication capacity. **b** Combinations of the Gag-Pro RC-decreasing mutations were generated on pNL4-3, and their effects on viral replication were investigated in vitro

We expanded our search for mutations in Gag that would affect Gag-Pro RCs in the B*52:01^−^ B*67:01^−^ subjects. In an exploratory analysis, we statistically identified amino acids in Gag that were associated with Gag-Pro RCs in persons lacking the protective B*52:01 and B*67:01 alleles. We identified amino acid variants at four positions, Gag 79, 228, 286, and 357 that were associated with lower Gag-Pro RC and at one position, Gag 218, which was associated with a higher Gag-Pro RC (Table 3). To evaluate the potential combinatorial effects of these mutations on Gag-Pro RC, we assigned a score to each recombinant Gag-Protease viral sequence by assigning +1 for the presence of the RC-increasing residue and −1 for each of the four RC-decreasing residues and computing this sum. Overall, these scores exhibited a significant positive correlation with Gag-Pro RCs (*R* = 0.35, *P* = 0.03 × 10^−6^; Fig. 4a). This suggests a dose-dependent effect of these substitutions on replication capacity, which we sought to confirm in vitro. To do this, we focused on the amino acid changes that reduced Gag-Pro RC and introduced Gag Y79F and R286K substitutions (the most frequent variations at these positions; Additional file 4), along with Gag M228L into pNL4-3 by site-directed mutagenesis. NL4-3 naturally carries glycine at Gag357 and therefore naturally contains a residue other than S at this site (357G). Note that the combination of these four substitutions 79F+228L+286K+357G was observed in naturally occurring sequences from our cohort. The mutant NL4-3 carrying these four Gag-Pro RC-reducing changes exhibited 20 % decreased replication capacity compared to wild-type (*P* < 0.0001; Fig. 4b). Thus, the in vitro result confirmed the effect of the amino acid changes on the viral replication capacity.

**Table 3:** Amino acid polymorphisms affecting viral replication capacities in the B*5201^−^/B*6701^−^ population

B52^−^ B67^−^ n = 218	Amino acid position		Amino acid		RC	Sample number		Median RC		Mann–Whitney U test	
			Consensus	Variant		aa+	aa−	aa+	aa−	*P* value	q value
Gag	79	Matrix	F (Y)^a^	Y	Lower	147	71	1.10	1.13	0.0081	0.18
	218	Capsid	V	V	Higher	58	160	1.13	1.10	0.028	0.26
	228	Capsid	M	L	Lower	12	206	1.03	1.11	0.015	0.22
	286	Capsid	K (R)^a^	R	Lower	115	103	1.09	1.13	0.029	0.26
	357	Capsid	G	S	Lower	159	59	1.10	1.16	0.0074	0.18

*aa+* sequences containing the amino acid variant in question, *aa−* sequences lacking the amino acid variant in question

^a^Consensus amino acids indicate those determined for the Japanese subjects. The amino acids in parentheses indicate clade B consensus

**Fig. 5:**
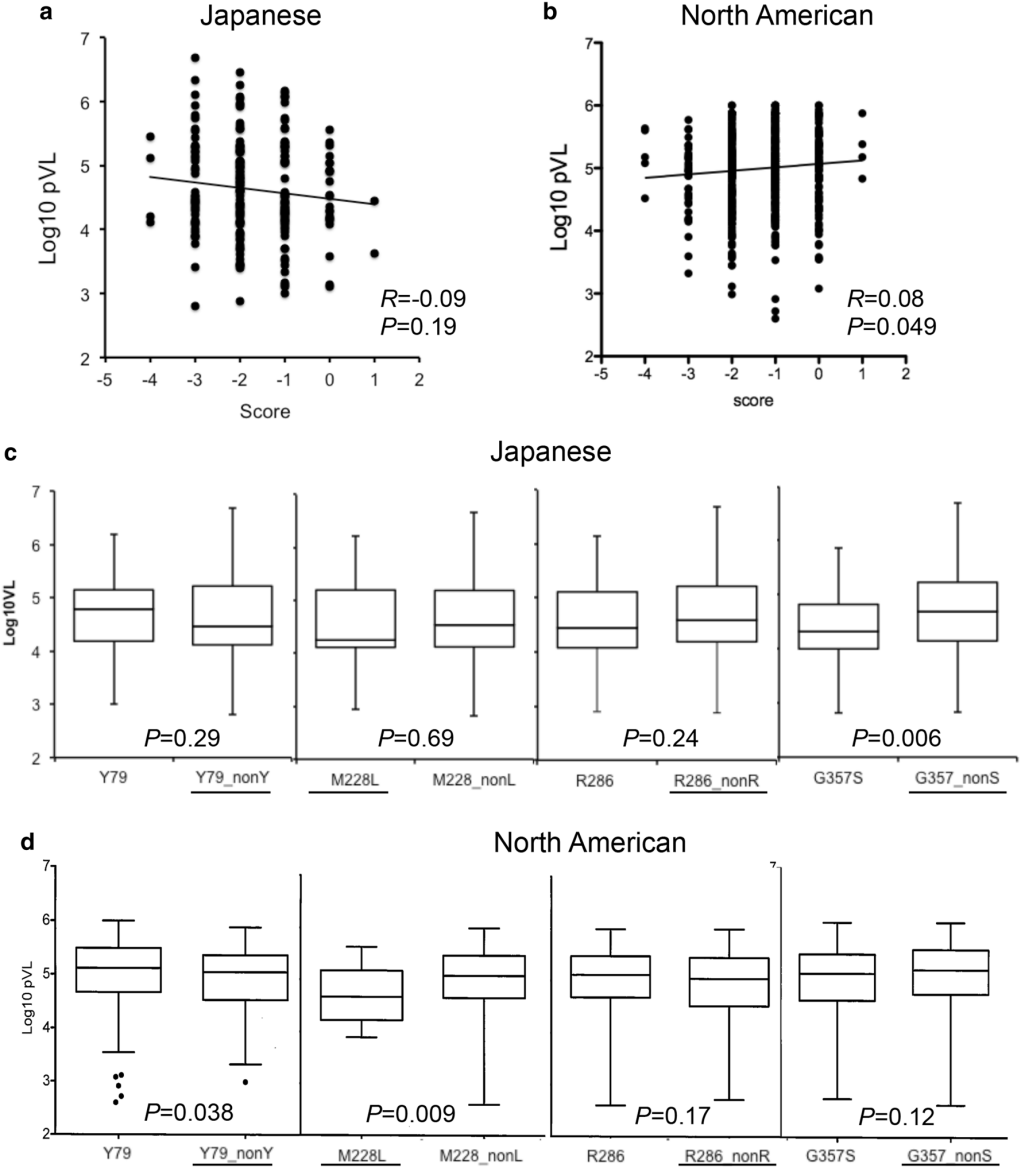
The impact of the amino acid polymorphisms on pVL. The x-axis indicates mutation scores as in Fig. 4. The y-axis represents pVL. Correlations between pVL and mutation scores were tested by Spearman’s rank correlation test for the HLA-B*52:01^−^B*67:01^−^ Japanese (**a**) and North American (**b**) cohorts described in [18]. **c**, **d** The effect of mutations at each position on pVL was examined in the HLA-B*52:01^−^B*67:01^−^ Japanese (**c**) and the North American (**d**) populations by the Mann–Whitney U test. The *underlined* samples indicate the presence of amino acid changes that reduce Gag-Pro RC

Even though the presence of these amino acid changes in the HLA-B*52:01^−^B*67:01^−^ subpopulation reduced viral replication capacity in vitro, they did not significantly correlate with pVL in this subpopulation (Fig. 5a). To investigate the potential in vivo importance of these amino acid changes in a non-Japanese cohort, we analyzed associations between these variants and pVL in a North American cohort that previously reported stronger positive correlations between Gag-Pro RC and pVL [18]. In the North American cohort, B*52:01/C*12:02 prevalence is <2 % and B*67:01 prevalence is 0 %, and thus these alleles are unlikely to influence the present analysis [18]. In contrast to the Japanese cohort, the North American cohort showed a weak association between pVL and mutation scores (*R* = 0.078, *P* = 0.049; Fig. 5b), suggesting a minor but significant impact of these polymorphisms in vivo. Individual analysis of mutations at each position also revealed significantly lower pVL among individuals harboring amino acid variants at Gag 79 (Y79 versus non-Y) and 228 (M228L) in the North American cohort (Fig. 5d). In contrast, no such relationships were observed in the HLA-B*52:01^−^B*67:01^−^ Japanese subpopulation (Fig. 5c). The observation that Gag amino acid variants, at least at position 79 and 228, are associated with pVL in North America but not Japan further supports a lesser role of Gag fitness in modulating clinical outcomes in Japan, even in the HLA-B*52:01^−^B*67:01^−^ subpopulation.

## Discussion

Mounting evidence suggests the importance of immune responses against Gag in the control of HIV-1 infection [reviewed in 12, 13]. In persons expressing protective HLA-B*57:01 and B*27:05 alleles, targeting critical immunodominant epitopes in Gag slows disease progression. In vitro viral replication studies, notably those using recombinant viruses carrying patient-derived Gag-Protease sequences, have contributed to this understanding by demonstrating significantly lower Gag-dependent viral replication in HIV-1 controllers from North American cohorts [25, 26]. Positive correlations between Gag-mediated replication capacity and pVL have also been reported in HIV-1 subtype C in an African cohort [28, 29] and subtype B in Mexican and Barbadian cohorts [30] which indicated an association between Gag-Pro RC and the frequency of protective HLA alleles in the population.

In contrast, our results indicate no significant association of Gag-Pro RC with pVL and CD4 in the overall Japanese cohort even though identical experimental methods were employed. This lack of significant correlation between Gag-Pro RC and pVL, possibly even in the B*52-B*67- subpopulation according to the multivariate analysis, suggests that Gag fitness does not substantially affect pVL in Japan and raises the hypothesis that Gag-mediated HIV-1 control is less critical in Japan than other populations. The difference could be due to the unique HLA distribution in Japan [31], which shapes viral diversity at a population level [1]. Particularly, the protective HLA-B*57:01 and B*27:05 alleles are virtually absent in Japan; instead, HLA-B*52:01 (allele frequency 10.4–11.1 %) and Asian-specific B*67:01 (allele frequency 1.1–1.7 %) are associated with HIV-1 control [Allele Frequencies database (http://www.allelefrequencies.net/default.asp); 32]. Intriguingly, neither B*52:01 nor B*67:01 was associated with low Gag-Pro RCs, and Gag-Pro RC did not correlate with clinical parameters in subjects with these protective alleles. As such, we hypothesize that the replicative consequences of B*52/B*67-driven immune escape mutations in Gag are minimal compared to the replicative effects of escape from protective alleles such as B*27 and B*57 in other populations. In support of this hypothesis, our previous results indicate that the number of HLA-associated amino acid changes in Pol, but not in Gag, negatively correlate with pVL [31]. The correlation was observed in the presence of HLA-B*52:01, suggesting that escape from HLA-B*52:01-restricted responses in Pol may confer fitness costs.

The absence of HLA-C*12:02, an allele in tight linkage disequilibrium with HLA-B*52:01, also showed significant associations between RC and both clinical parameters. Our previous study demonstrated a negative correlation between pVL and B*52:01-associated amino acid changes in Pol whereas C*12:02-associated changes did not show a significant association [31]. However, the effect of B*52:01 cannot be separated in the present study due to the extremely low number of B*52:01 or C*12:02 single positive subjects. The presence of C*08:01 (n = 34) and C*08:03 (n = 10) also had strong associations with both clinical parameters, but these two alleles are not significantly associated with lower or higher pVL in chronic phase in our previous study [32]. Therefore, the clinical importance and a putative role of these associations require further investigation.

We identified amino acid polymorphisms in Gag that were associated with alterations of Gag-Pro RC in the absence of protective HLA-B*52:01 and B*67:01 alleles. Of the four polymorphisms associated with lower RC, one was located in p17 (Y79) and the other three in p24 (M228, R286, G357). Our previous analysis of HLA-associated viral polymorphisms in this cohort indicated that changes at Y79 were associated with HLA-B*51:01, C*14:02, and C*14:03, changes at M228 were associated with C*0304, and changes at G357 were associated with B*07:02 [31]. Of note, R286 had been previously identified as B*52:01-associated even though B*52-expressing subjects were excluded from the present analysis of RC-associated substitutions [31]. It is therefore possible that R286K imposes a fitness cost in B*52:01^+^ subjects, but its effect on Gag-Pro RC may have been indiscernible due to the presence of other RC-modulating mutations in these individuals. Moreover, the impact of R286K on pVL among B*52:01 individuals may be difficult to detect due to two conflicting forces at play: on one hand, R286K may slightly lower replication capacity, but on the other hand, it confers the ability to escape from CTL responses and thus persist in vivo. For B*52:01^−^ subjects, the B*52-driven R286K immune-escape mutation may have been transmitted to B*52:01^−^ recipients, which may have resulted in the observed lower Gag-Pro RC (similar to the reduced RCs observed when the B*57:01-restricted T242N is transmitted to B*57:01^−^ recipients [8, 9] ). Site-directed mutagenesis confirmed that the four amino acid changes significantly reduced Gag-Pro RC. However, the reduction in Gag-Pro RCs by these amino acid changes did not lead to lower pVL even in the B*52^−^B*67^−^ subpopulation, suggesting a possible lack of a significant impact of Gag fitness on pVL in Japan.

Even though our data argue against a significant role of Gag fitness as a major determinant of HIV-1 disease outcome in Japan, they do not completely exclude the possibility that Gag fitness subtly influences pVL. The amino acid changes may have significantly decreased pVL, but other mutations in viral genome could have restored viral replication capacity in vivo. These scenarios still suggest that pVL does not reflect change in Gag fitness, and that Gag replication has only a minimal impact on HIV-1 pathogenesis in Japan. Further studies, including longitudinal analyses, will be necessary to identify such compensatory mutations. Alternatively, HLA-associated polymorphisms in Gag could actually be the result of immune pressure. In this case, a high immune pressure in Gag would generate immune-escape mutations, but the reduction of pVL may have occurred through cross-reactive CTL function rather than mutation-induced Gag fitness cost. A recent functional study on CTL responses reported 13 epitopes (eight in Gag and five in Pol) involved in HIV-1 control in Japan [39]. B52* and B*67 targeted eight out of the 13 epitopes, including seven of the eight epitopes located in Gag, suggesting the importance of the Gag as an immune target by the Japanese protective alleles [39]. Surprisingly, however, only one of the eight disease-controlling CTL epitopes targeted by B*52 induced escape mutations [39]. These results suggest that CTL responses directly control pVL by killing infected cells via HLA-mediated recognition of Gag epitopes, rather than via escape-mediated fitness costs. In addition to sustained responses to Gag epitopes that are difficult for the virus to escape [39], HLA-mediated protective effects in Japan may also be mediated by responses to Pol epitopes that eventually induce fitness-costly escape mutations within that viral protein, that partially offset the advantages gained by escape from CTL responses [31].

Gag fitness has an effect on CD4 count in the Japanese population who do not express protective alleles, in contrast to global cohorts. At least in this subpopulation, escape mutations in Gag may impose fitness costs that are clinically advantageous. However, viral polymorphisms that weakly affected pVL did not have an impact on Gag fitness even in this B*52^−^B*67^−^ subpopulation, implying a minor contribution of Gag fitness to HIV-1 clinical outcome. Fitness costs of immune-driven mutations in other viral proteins may have more significant impact on HIV-1 disease progression in Japan [31]. These observations are in contrast to the previous reports which indicated significant correlations between Gag-Pro RCs and clinical parameters in Mexican/Barbadian and African chronic cohorts as well as elite controllers and subjects carrying protective HLA alleles in predominantly Caucasian chronic cohorts [18, 25, 27–30].

Previously, one study analyzed Gag-Pro RC in 156 treatment-naive Japanese individuals sampled from 1994 to 2009 and reported reduced Gag-Pro RCs among more recent isolates [40]. In contrast to our results, they observed a significant association between Gag-Pro RC and pVL. The discrepancy between these two studies may be due to the characteristics of the cohorts and/or study time course. The present study analyzed samples collected from 2008 to 2011 whereas the previous one focused on older samples. Newer viruses may have more chance to accumulate mutations that could affect viral replication dependently or independently of those occurring in Gag-Protease. Mutations located outside of Gag-Protease could affect pVL but not Gag-Pro RCs measured in vitro, which would reduce the correlation between Gag-Pro RC and pVL. Sequence comparisons identified significantly different amino acid frequencies at 35 out of 500 amino acid positions in Gag and eight out of 100 positions in Pol between the two cohorts, which may have affected different outcomes in the two cohorts (data not shown). In addition, cohort-level differences in HLA prevalence (e.g. in the other study, the frequencies of A*24^+^ and C*03^+^ individuals were 52.6 and 34.6 %, respectively whereas the frequencies of these alleles in the present study were 62.4 and 42.2 %, respectively) may also have affected the results.

Notably, an association between Pol replication capacity and HIV-1 disease progression has also been reported in North American cohorts [41–46]. One of these studies suggested decreased reverse transcriptase-integrase-dependent RC in HIV-1 elite controllers [41]. In another study B*52:01-associated change at amino acid I220 in integrase was shown to decrease viral replication capacity [42]. Considering that Pol enzymatic functions directly affect viral replication, mutations in Pol may meaningfully impact viral fitness and pVL. Possibly, HLA-associated HIV-1 polymorphisms in Gag-Protease may not affect overall HIV-1 disease outcome in Japan as only a few alleles, including HLA-C*08:01 and C*08:03, had relatively strong correlations between Gag-Pro RC and clinical markers of infection. Additionally, the present study comprised a cross-sectional analysis at the chronic phase of HIV infection as previously done in the other global cohort studies except for the North American cohort. Thus, we cannot exclude a possibility of a significant impact of Gag fitness on clinical outcome at different time points: e.g. at the acute/early phase. Further study, including longitudinal analyses, will be necessary to determine a possible role of Gag fitness at the earlier phase as well as Pol fitness in HIV-1 disease progression in Japan.

## Conclusions

Our data support a lack of a significant impact of Gag fitness on pVL in treatment-naive Japanese individuals chronically infected with HIV-1. Moreover, in contrast to non-Asian chronic cohorts where Gag-Pro RC fitness may modulate HIV clinical parameters especially in subjects with protective HLA alleles, the protective effects of Japanese B*52 and B*67 alleles do not seem to be associated with Gag fitness. Thus, the impact of Gag fitness on HIV clinical parameters is population dependent.

## Methods

### Study subjects

A total of 430 treatment-naive Japanese subjects chronically infected with HIV-1 subtype B were recruited for a previous study [31] and enrolled at the National Center for Global Health and Medicine (NCGHM) from 2008 to 2011. HLA typing and the preparation of cDNA samples from patient plasma were performed as described in the previous study. For the present study, specimens from 306 of the original 430 participants were randomly selected for analysis. The median pVL and CD4 count for this N = 306 cohort were 4.5 log_10_ RNA copies/ml (IQR: 4.1–5.1) and 299 cells/mm^3^ (IQR: 151–407), respectively. The study was approved by the Ethical Committees in the Faculty of Life Science, Kumamoto University and the NCGHM and conducted in accordance with the Declaration of Helsinki.

For comparison with the Japanese cohort, a data set derived from a published North American (Canada and USA) cohort comprising 803 untreated chronic subjects infected with HIV-1 subtype B was used for the analysis [18]. Median pVL and CD4 count for the cohort were 5.1 log_10_ RNA copies/ml (IQR: 4.7–5.5) and 273 cells/mm^3^ (IQR: 130–420), respectively [18].

### Generation of chimeric viruses and in vitro replication assays

The production of chimeric viruses and Gag-Pro RC determination was performed as described previously [18, 25–30]. Briefly, cDNAs were generated from plasma viral RNA for a separate study [31]. Plasma-derived *gag*-*protease* DNA fragments were generated from the cDNAs by nested PCR, using TaKaRa ExTaq Hot Start Version (Takara Bio Inc., Shiga, Japan). Chimeric HIV-1_NL4-3_ encoding plasma-derived *gag*-*protease* was generated by electroporating *gag*-*protease* PCR fragments with linearized *gag*-*protease*-deficient pNL4-3 (*delta gag*-*pro* NL4-3) into a CEM-derived GFP-reporter T-cell line engineered to carry LTR-GFP, CXCR4, and CCR5 (CEM-GXR, a generous gift from T. Miura and M.A. Brockman) [38] . Stock titers were determined by infecting CEM-GXR and measuring GFP^+^ cells, using flow cytometry. To determine Gag-Pro RCs, CEM-GXR cells were infected with chimeric viruses at a MOI of 0.03 at day 2, and the percentage of HIV-infected (GFP-expressing) cells was monitored daily for 2–8 days post-infection. Gag-Pro RC was calculated as the natural-log slope of the percentage of GFP-expressing cells during the exponential phase of viral spread and normalized against the mean value obtained with wild-type NL4-3 (i.e. NL4-3 RC = 1.00). Virus titration and RC experiments were done in triplicate.

For the validation of virus stocks, we isolated RNA from all virus stocks, using EZ1 Virus Mini Kit v2.0 (Qiagen, Valencia, CA). *Gag*-*protease* DNA fragments were generated by RT-PCR, using SuperScript III First-Strand Synthesis System for RT-PCR (Life Technologies, Carlsbad, CA) for cDNA synthesis and TaKaRa ExTaq Hot Start Version for subsequent nested PCR. Viral sequences were analyzed on the ABI3500 genetic analyzer (Applied Biosystems, Carlsbad, CA) and compared with previously determined *gag*-*protease* sequences from patients’ plasma viruses [31] by constructing a maximum-likelihood phylogenetic tree (PhyML) [47]. Phylogenies were viewed using Figtree (http://tree.bio.ed.ac.uk/software/figtree).

### Definition and analysis of HLA-associated polymorphisms in Gag-Protease

A total of 110 HLA-associated polymorphisms, occurring at 61 unique codons in Gag-Protease, were previously defined in a cohort of 430 treatment-naive Japanese subjects using phylogenetically-corrected methods [31]. In the present study we wished to identify HLA-associated polymorphisms that modulate Gag-Pro RC. For this purpose, we compared Gag-Pro RCs in the presence or absence of each of these HLA-associated amino acid substitutions and assessed significance using the Mann–Whitney U test. Figures depicting amino acid variation at Gag-Pro-RC-modulating codons were created using WebLogo 3 (http://weblogo.threeplusone.com) [48, 49] to visualize sequence conservation at each position.

### Site-directed mutagenesis and mutant virus stocks

Y79F, M228, and R286K substitutions were engineered into HIV-1 pNL4-3 by PCR overlap extension [50, 51], using PCR primers containing specific nucleotide changes: *tat* to *ttc* for Y79F, *atg* to *ctg* for M228L and *aga* to *aag* for R286K. These specific triplet codons were selected for each substitution based on higher frequencies in human codon usage, to exclude possible negative effects of rare codon usage in vitro. PCR fragments containing specific nucleotide changes were digested with BssHII (at nucleotide position 711 in pNL4-3) and SbfI (at 2838 in pNL4-3) followed by subcloning into pNL4-3 digested with the same restriction enzymes. The subcloned fragments were sequenced to confirm specific nucleotide changes and the absence of other mutations.

pNL4-3 carrying specific mutations were transfected into HEK293T cells by lipofectamine 2000 (Life Technologies, Carlsbad, CA) according to the manufacturer’s instructions. Virus stocks were harvested 48 h post-transfection, and titers were determined by infecting CEM-GXR cells and subsequently measuring GFP^+^ cells. Replication assays were performed in two sets of triplicate for each sample as described above.

### Statistical analysis

Associations between Gag-Pro RC, clinical parameters and mutation scores were determined by Spearman’s rank correlation. Differences in pVL in context of the presence or absence of amino acid substitutions at four locations in Gag were determined by the Mann–Whitney U test. Multiple comparisons were addressed using q-values.

The relationship between Gag-Pro RC and pVL/CD4 count in the population subset that did not express protective HLA class I alleles (HLA-B*52:01^−^B*67:01^−^ subset) was also investigated using multivariable linear regression. The primary model included only log_10_ plasma viral load (per log_10_ increment) and CD4 count (per 1 cell/mm^3^ increment) as variables. A second model additionally incorporated specific HLA alleles (C*08:01, C*08:02, C*12:02) that exhibited significant associations between Gag-Pro RC and both pVL and CD4 count as variables; here, model estimates are expressed in terms of individuals harboring (versus the reference group of individuals not harboring) the HLA allele in question. Multivariable analyses were performed using R (version 3.1.2).

### Ethics statement

The study was approved by the Ethical Committees in the Faculty of Life Science Kumamoto University (540) and the National Center for Global Health and Medicine. Informed consent was obtained from all patients, and the study was conducted in accordance with the Declaration of Helsinki.

## Additional files

10.1186/s12977-015-0223-z Comparison of *gag*-*protease* nucleotide sequences from viral stocks and patients’ plasma viruses (total N = 306). (A) A maximum-likelihood phylogenetic tree was constructed for *gag*-*protease* sequences from all samples to verify their identities (n = 287). (B) The rest of 19 samples were analyzed separately due to the presence of gaps in the original sequences.
10.1186/s12977-015-0223-z Replication capacities for HLA alleles prevalent in Japan and their associations with clinical parameters. (A-C) All HLA alleles occurring at a phenotypic frequency of greater than approximately 3 % (n > 8) were examined for their associations with viral load. The median (white line), minimum and maximum values (whiskers), and interquartile range (box) were indicated. The black vertical lines indicate the median RC for the whole population (N = 298). HLA alleles were arranged from lowest (top) to highest (bottom) by their mean replication capacities. (D) The relationship between median Gag-Pro RCs and median pVL. Correlations were examined by Spearman’s rank correlation test. Each circle represents median Gag-Pro RC and pVL for a group of subjects expressing one of the HLA alleles.
10.1186/s12977-015-0223-z Associations of Gag-Pro RCs with clinical parameters in each HLA-class-I allele in the absence of B*52:01/B*67:01/C*12:02.
10.1186/s12977-015-0223-z The positions and variations of amino acid sequences involved in the reduction of Gag-Pro RCs in the HLA-B*52:01^−^/B*67:01^−^ population. The arrows indicate the positions of amino acid changes. Amino acid numbering below each sequence was based on the HIV-1 HXB2 strain. Consensus amino acids are indicated on the left and mutations on the right of each logo. Hydrophilic residues (RKDNQ) were shown in blue, neutral residues (SGHTAP) in green, and hydrophobic residues (YVMCLFIW) in black.

## Abbreviations

HLA: human leukocyte antigen
pVL: plasma viral load
RC: replication capacity
CTL: cytotoxic T lymphocytes
