# Erratum to: Lack of a significant impact of Gag-Protease-mediated HIV-1 replication capacity on clinical parameters in treatment-naive Japanese individuals

**DOI:** 10.1186/s12977-015-0227-8

**Published:** 2015-11-30

**Authors:** Keiko Sakai, Takayuki Chikata, Zabrina L. Brumme, Chanson J. Brumme, Hiroyuki Gatanaga, Shinichi Oka, Masafumi Takiguchi

**Affiliations:** Center for AIDS Research, Kumamoto University, Kumamoto, 860-0811 Japan; International Research Center for Medical Sciences, Kumamoto University, Kumamoto, 860-0811 Japan; British Columbia Centre for Excellence in HIV/AIDS, Vancouver, BC Canada; Faculty of Health Sciences, Simon Fraser University, Burnaby, BC V5A 1S6 Canada; National Center for Global Health and Medicine, Tokyo, 162-8655 Japan; Nuffield Department of Medicine, University of Oxford, Oxford, UK

## Erratum to: Retrovirology (2015) 12:98 DOI 10.1186/s12977-015-0223-z

The original version of this article [1] unfortunately contained a mistake. In the author list, the surname of author Hiroyuki Gatanaga was incorrectly spelled. The original article has now been updated with the correct spelling.

## References

[1] Sakai K, Chikata T, Brumme ZL, Brumme CJ, Gatanaga H, Oka S, Takiguchi M (2015). Lack of a significant impact of Gag-Protease-mediated HIV-1 replication capacity on clinical parameters in treatment-naive Japanese individuals. Retrovirology.

