# Supplementary figures and images for: Lack of a significant impact of Gag-Protease-mediated HIV-1 replication capacity on clinical parameters in treatment-naive Japanese individuals
Source: Retrovirology. 2015 Nov 19;12:98. doi: 10.1186/s12977-015-0223-z (PMC4653850; doi:10.1186/s12977-015-0223-z)

A

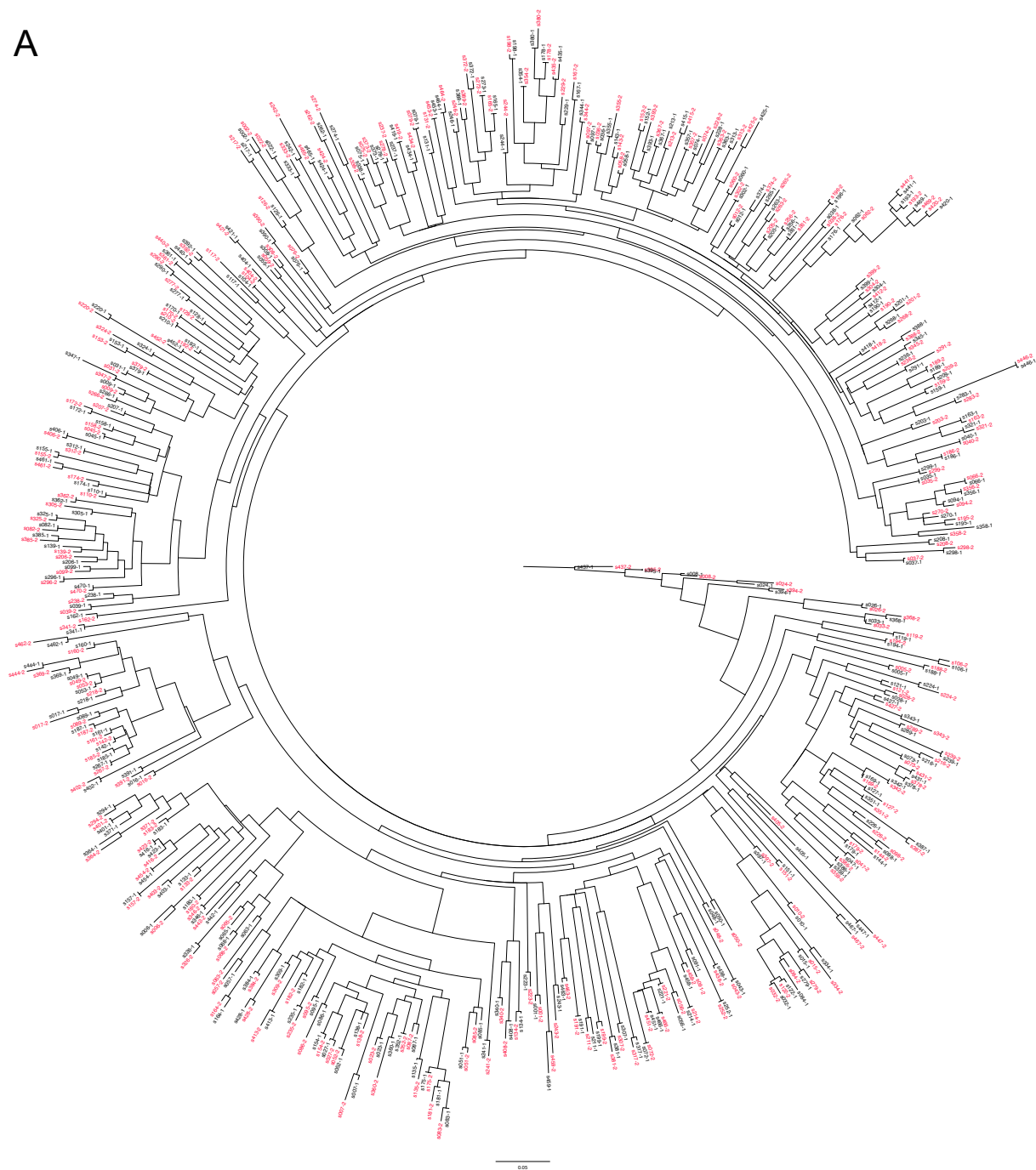

B

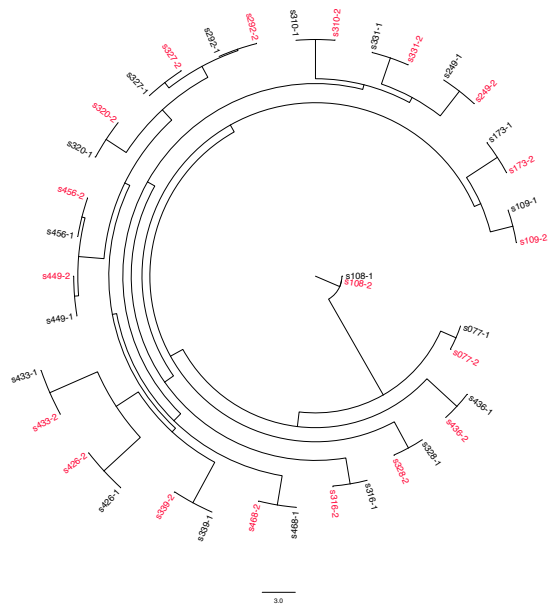

Supplement: Supplementary file 1 — 10.1186/s12977-015-0223-z Comparison of gag-protease nucleotide sequences from viral stocks and patients’ plasma viruses (total N = 306). (A) A maximum-likelihood phylogenetic tree was constructed for gag-protease sequences from all samples to verify their identities (n = 287). (B) The rest of 19 samples were analyzed separately due to the presence of gaps in the original sequences. [file 12977_2015_223_MOESM1_ESM.pdf]

**A**

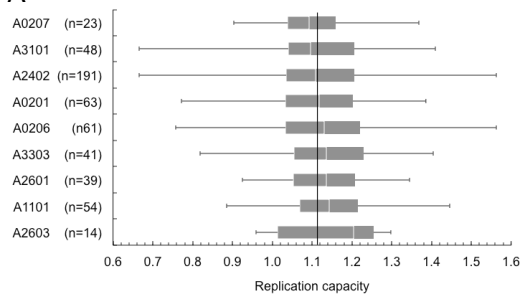

**B**

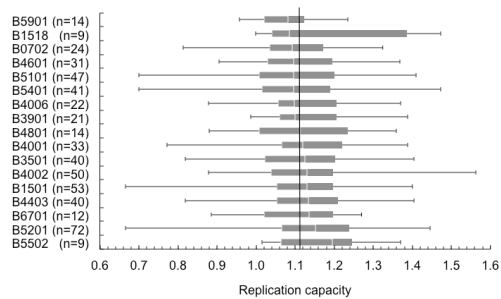

**C**

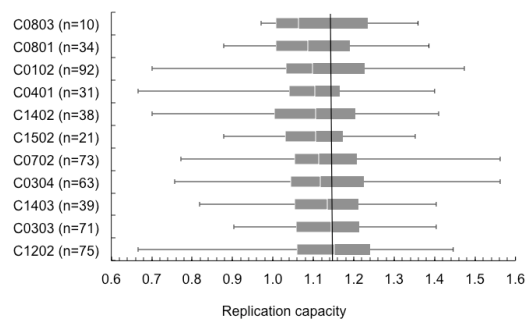

**D**

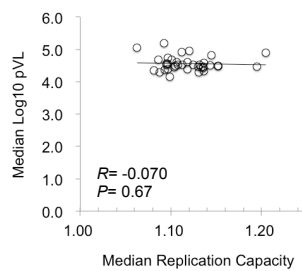

Supplement: Supplementary file 2 — 10.1186/s12977-015-0223-z Replication capacities for HLA alleles prevalent in Japan and their associations with clinical parameters. (A-C) All HLA alleles occurring at a phenotypic frequency of greater than approximately 3 % (n > 8) were examined for their associations with viral load. The median (white line), minimum and maximum values (whiskers), and interquartile range (box) were indicated. The black vertical lines indicate the median RC for the whole population (N = 298). HLA alleles were arranged from lowest (top) to highest (bottom) by their mean replication capacities. (D) The relationship between median Gag-Pro RCs and median pVL. Correlations were examined by Spearman’s rank correlation test. Each circle represents median Gag-Pro RC and pVL for a group of subjects expressing one of the HLA alleles. [file 12977_2015_223_MOESM2_ESM.pdf]

consensus

mutations  
generated

Y79

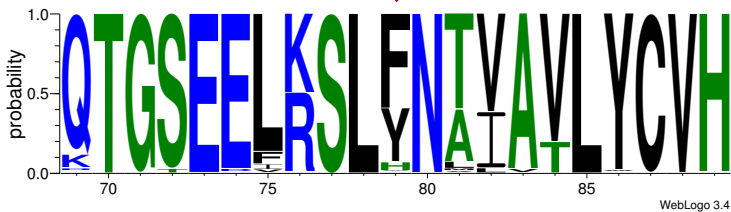

Y79F

M228

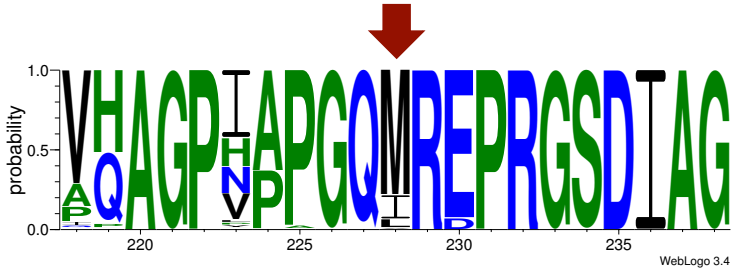

M228L

R286

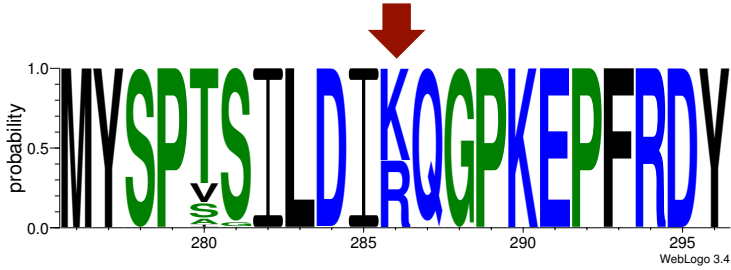

R286K

G357

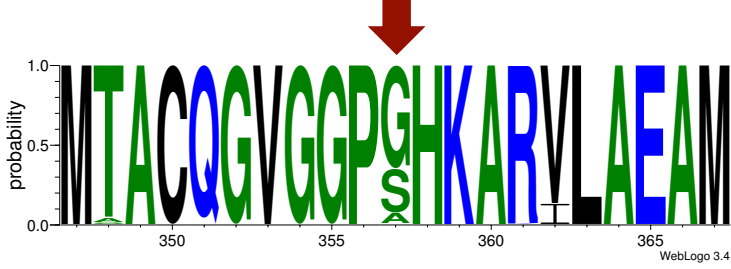

G357S

Supplement: Supplementary file 4 — 10.1186/s12977-015-0223-z The positions and variations of amino acid sequences involved in the reduction of Gag-Pro RCs in the HLA-B*52:01−/B*67:01− population. The arrows indicate the positions of amino acid changes. Amino acid numbering below each sequence was based on the HIV-1 HXB2 strain. Consensus amino acids are indicated on the left and mutations on the right of each logo. Hydrophilic residues (RKDNQ) were shown in blue, neutral residues (SGHTAP) in green, and hydrophobic residues (YVMCLFIW) in black. [file 12977_2015_223_MOESM4_ESM.pdf]
