# Supplementary material for: Lack of a significant impact of Gag-Protease-mediated HIV-1 replication capacity on clinical parameters in treatment-naive Japanese individuals
Source: Retrovirology. 2015 Nov 19;12:98. doi: 10.1186/s12977-015-0223-z (PMC4653850; doi:10.1186/s12977-015-0223-z)
Supplement: Supplementary file 3 — 10.1186/s12977-015-0223-z Associations of Gag-Pro RCs with clinical parameters in each HLA-class-I allele in the absence of B*52:01/B*67:01/C*12:02. [file 12977_2015_223_MOESM3_ESM.pdf]

Additional file 3: Table S1

Associations of Gag-Pro RCs with clinical parameters in each HLA-class-I allele in the absence of B*52:01/ B*67:01/ C*1202.

HLA allele	HLA + or -[a]	n[b]	R[c]	*P* value	HLA allele	HLA + or -[a]	n[b]	R[c]	*P* value
**A*11:01**	-	185	0.20	0.0057	**A*11:01**	-	185	-0.22	0.0025
**A*33:03**	-	188	0.25	0.0006	**A*33:03**	-	188	-0.24	0.0012
**B*39:01**	-	202	0.22	0.0020	**B*39:01**	-	202	-0.24	0.0006
**B*44:03**	-	187	0.25	0.0007	**B*44:03**	-	187	-0.23	0.0021
**B*52:01**	-	221	0.15	0.0224	**B*52:01**	-	221	-0.23	0.0007
**C*07:02**	-	171	0.21	0.0052	**C*07:02**	-	171	-0.22	0.0041
**C*12:02**	-	221	0.18	0.0094	**C*12:02**	-	221	-0.22	0.0009
**C*14:03**	-	188	0.25	0.0007	**C*14:03**	-	188	-0.23	0.0017

[a]HLA+ and HLA- indicate subjects with or without the particular HLA allele, respectively.

[b]n: number of subjects

[c]R: Spearman's rank correlation

[d]Bold HLA alleles have associations with both pVL and CD4 count.
